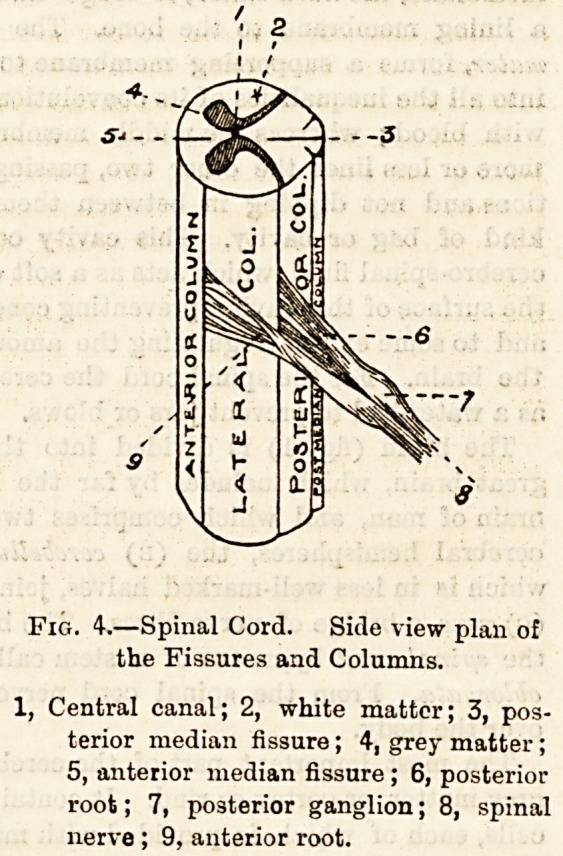# The Hospital. Nursing Section

**Published:** 1903-11-28

**Authors:** 


### The Hospital.

### Nursing Section.

Contributions for this Section of "The Hospital" should be addressed to the Editor, "The Hospital" Nursing Section, 28 & 29 Southampton Street, Strand, London, W.C.

No. 896.—Vol. XXXV. *SATURDAY, NOVEMBER* 28, 1903.

### Notes on News from the Nursing World.

### OUR CHRISTMAS DISTRIBUTION.

Among the applications which we have received for a share in our Christmas distribution is one from the matron of an East End hospital who has a ward containing 22 children of all ages, and she expresses a hope that if we find it impossible to send a few toys, "perhaps one or two garments for the adult patients might be spared." We do not profess to distribute toys—perhaps the Editor of *Truth* may be able to assist—but we shall probably be able to respond to the other portion of the appeal. As we are now within three weeks of the time when the articles will be sent to the hospitals and infirmaries, we ask our readers to forward their parcels as soon as possible, addressed to the Editor, 28 and 29 Southampton Street, Strand, London, W.C., with "Clothing Distribution" written outside them. We have to acknowledge contributions as follows :—Miss E. Farrer, Godley, Cheshire ; R.N.P.F., 3521 ; Nurses B. and A. Hillman, Charmouth ; Nurse Cave-Browne-Cave, Stillington, Easingwold ; and a contribution without the name of the sender.

### QUEEN ALEXANDRA AND DISTRICT NURSES.

At the annual meeting of the Warrington District Nursing Association last week, many tributes were paid to the Queen's nurses by people competent to judge of the value of their work. Three reports were adopted, namely, those of the committee, the superintendent, and the treasurer. The first offered the warmest thanks of the committee to the superintendent and the nurses for their untiring devotion to their duties ; in the second a summary was given which showed the number of cases under notice to have been 492, and the number of visits paid 17,567 ; and the third contained the satisfactory announcement that after paying all expenses, there was a credit balance of £44. The chairman, in his speech, mentioned two gratifying evidences of the popularity of the organisation in the town. Thus, the local anglers sent in a contribution of £26 a few days ago, and to a performance on the part of the St. Mary's Dramatic Society the Association was indebted for a considerable sum. Miss Whitfield, the superintendent, in acknowledging a vote of thanks to the nurses, said that such a vote was quite unnecessary. "Several years ago," she continued, "when they were invited to Marlborough House, Queen Alexandra gave them a little address and in a few of her words she exactly expressed the feeling of the nurses. Her Majesty said, 'I can, indeed, imagine no higher nor holier calling than that of being able to tend the poor and suffering in the hour of their greatest need.'" We are sure that Queen Alexandra will be gratified to learn how her words have been taken to heart.

### THE MATRON OF JOHANNESBURG HOSPITAL AND HER NURSES.

The new matron of Johannesburg Hospital has, rightly or wrongly, very soon come into collision with her nurses. From the various newspaper cuttings which have reached us we learn that the Board of the hospital have, so far, justified the course pursued by the matron as to pass unanimously, after a long inquiry, a resolution bidding her to severely reprimand the nursing staff and threatening them that if there should be any more trouble with them they will be dealt with summarily. There are some points, however, which seem not to have been cleared up. The inquiry of the Board was undertaken ac the instance of the nurses, who charged the matron with an attack on their character, and the matron herself does not dispute that in reproving the probationers for their behaviour in the hospital grounds after dark she reminded them "of the fate which befell a certain notorious female mentioned in the Book of Genesis." The matron states that her words and meaning had been misunderstood ; and the Board, in reprimanding the nurses, might have suggested to the matron that her Biblical analogy was unfortunate. It has since been alleged on behalf of the nurses as reasons for their attitude of hostility to the matron that she insists upon their doing a stretch of twelve months' night duty, that she does not permit them to receive relatives or friends in the nurses' quarters, that the nurses' bedrooms are overrun by rats, that a large number of nurses are now lodged in iron shanties which become unbearably hot in the summer, and that the number of nurses put on night duty is often wholly inadequate. It seems to us a pity that the nursing staff at the Johannesburg Hospital, who have been found guilty by the Board of a breach of discipline, did not take the opportunity of laying stress on grievances which, if reports are correct, are very real.

### THE LIABILITY OF NURSING ASSOCIATIONS FOR THE SICK POOR.

The result of the action against the committee of the Oldham Nursing Association has been followed in several local and other papers by a correspondence, and some very foolish comments have been made upon the decision. For example, a member of another nursing organisation declares that in view of the damages awarded against the Oldham association, "it behoves those who have been instrumental in organising similar charities to seriously consider their situation," and he contends that "some means for limiting the liability of persons engaged in a very useful work should be found." But even people who are engaged in a very useful work must expect to take a measure of risk. At all events, it is quite out of the question that any nursing association which tries to perform the dual task of ministering to the sick poor and employing its nurses in private cases should be allowed, while pocketing the nursing fees, to escape responsibility for the mistakes of its salaried servants.

### DOMESTIC ECONOMY AND NURSING.

A sensible letter appears in another column from an Irish superintendent nurse in a workhouse infirmary. Having pointed out that in Ireland the superintendent nurse has for some years past been independent of the workhouse master and matron, and subject only to the medical officer, she urges that the Poor-law service is eminently adapted to older nurses who have ceased to appreciate the constant excitement of interesting cases and the consequent wear and tear of hospital life. We gather from the account of her own experiences that there are openings in the sister island for the considerable class which she claims to represent. She mentions, however, that it is practically as important for a superintendent nurse in an Irish workhouse infirmary to thoroughly understand and be able to enforce domestic economy, as it is for her to be fully trained. There is no doubt that a thorough knowledge of domestic economy is becoming increasingly indispensable to all nurses who aspire to fill posts of responsibility. It is therefore with satisfaction that we learn that the Croydon guardians have adopted a report of the infirmary committee, recommending a course of not less than 18 lectures on sick cookery to the nurses in their infirmary.

### OPPOSITION TO A RESIDENTIAL CLUB IN MELBOURNE.

The proposal of Miss Glover to establish a residential club ruled by a committee, has provoked a remarkable amount of opposition. No fewer than 153 members of the Victorian Trained Nurses' Association have emphatically protested against the scheme, and at a meeting of the council a letter was read from the matron of Bendigo Hospital, in which she observed that the whole project was so preposterous and would require such a tremendous outlay, that after reading the proposal she did not give it a second thought. In the course of her letter Miss Farquharson points to the unfortunate position of the London Co-operation, which, she says, entirely fails to give its members satisfaction, and adds: "Wide and deep are the discussions between the nurses and their committee, and an excellent lady superintendent resigned not long ago because of the management not being in the best interests of nurses." Miss Farquharson concludes with some wise words of warning: "I think it the duty of all senior members of the profession to urge on our younger sister nurses the absolute necessity of economy, avoidance of extravagance and debt, and putting by for the inevitable rainy day and old age, either by Savings Bank account or the Royal National Pension Fund for Nurses. But if they launch out into semi-luxurious residential clubs, there will be only poverty and ruin to look forward to." There is every appearance from the comments of the president of Victoria Trained Nurses' Association in Miss Farquharson's letter that the scheme will not be proceeded with; and in face of the hostility it has encountered, Miss Glover herself may determine to drop it. But we may observe that the success or failure of government by committee depends entirely upon the constitution of the latter If the Nurses' Co-operation in this country had always been governed by a strong and tactful committee, the criticisms of Miss Farquharson respecting it would have been impossible.

### A NURSE EXPECTED TO DINE WITH A STABLEMAN.

The difficulty of getting nurses to remain for any length of time in some of the country union infirmaries is a standing topic at meetings of Boards of Guardians. At a meeting of the Chertsey Board of Guardians last week, the case of a nurse who had only recently been appointed to the position of staff nurse sending in her resignation on the ground that she was looking out for a more suitable appointment was discussed. It was stated that the nurse, highly qualified and trained, had objected to the mess-room arrangements, where she had to take her meals with the stableman, who looked after the horses. She had not made any actual complaint, only she did not like it. Some of the Guardians considered the objection quite reasonable. It was also pointed out that the fact of the stableman having his meals in the room had been mentioned more than once. Eventually, it was decided that this extraordinary state of things should be stopped and the resignation was accepted.

### POOR-LAW INSPECTORS AND NURSING.

Less than usual prominence is given in the thirty-second annual report of the Local Government Board to workhouse nursing. Mr. Hervey, inspector for the district comprising the Union Counties of Norfolk and Suffolk, and parts of Essex and Cambridge, says that "the nursing is slowly but surely improving," and he mentions that two boards of guardians have seen the necessity for appointing superintendent nurses. Mr. Fleming, inspector for the district comprising the Union Counties of Dorset and Southampton, and parts of Wilts and Surrey, observes that "the position of the nursing question in the district generally has maintained its improvement, though there are still workhouses where it is far below the right standard." Mr. Wethered, inspector for the district comprising the Union County of Gloucester, and parts of Hereford, Somerset, Stafford, Wilts, and Worcester, merely gives a table showing that the number of nurses in proportion to patients is now one nurse to 14 patients. Mr. Jenner-Fust, inspector for the district comprising the Union Counties of Lancaster and Westmorland, together with part of Cumberland, affirms that the necessary return "maintains its satisfactory character." He mentions that out of 891 probationers trained in 1902 in 13 of the largest workhouse infirmaries in Lancashire, 118 remained in the service of the guardians after completing their training, and 139 obtained Poor-law appointments elsewhere. Of the rest 164 obtained posts in general hospitals—a fact to be noted by infirmary-trained nurses who protest against their general-hospital trained sisters entering the Poor-law service—53 were engaged as Queen's nurses, 258 as private and district nurses, 12 returned to Poor-law service after other employment, 59 left to be married, and 68 are unaccounted for. Mr. Bagenal, inspector for the Union Counties of the East and West Ridings of Yorkshire, states that "the only unsatisfactory feature of the nursing return is that in 22 workhouses in the district there is no nurse on duty at night." Finally, Mr. James Lowry, inspector for the Union Counties of Northumberland, Durham, the North Riding of Yorkshire, and part of Cumberland, reports that "quite a number of the unions in the district have lately increased their nursing staff."

### IS A NURSE AN INHABITANT?

A dispute of considerable interest to nurses in the service of public bodies has arisen in Warrington. The matter came up as a result of a nurse in the service of the Board of Guardians falling ill with scarlet fever. The Board wished to send the nurse to the Corporation's isolation hospital. The authorities refused unless payment were made. Correspondence ensued between the clerk to the Guardians and the Town Clerk, in which the former pointed out that the nurse was an inhabitant of the municipal borough just as much as any officer of the Board who might live outside the Workhouse. The Workhouse paid rates on an assessment of £850, and the whole of the hospital charges of the borough were included in the rates, so that the Guardians were paying towards the cost of maintaining patients in the fever hospital, whether rich or poor. There the matter stands for the present. It is expected that the Local Government Board will decide the question at issue.

### THE NURSES OF TORONTO GENERAL HOSPITAL.

At the year's graduating exercise of the Toronto General Hospital School for Nurses last month, a score of graduates received their certificates. Before the presentation, the twenty-second annual report of the school was read, and it showed that during the year there had been a considerable increase in the demand for special and dispensary nurses. In addition to the work of the regular staff of nurses, there were 1,625 days of special nursing in the 12 months. In addition to the badges and diplomas given to the nurses receiving certificates, handsomely-bound volumes were also handed to the three nurses who had taken the highest standing. After the exercises a reception was held in the Nurses' Home.

### NURSES' MISSIONARY UNION.

The secretary of the Nurses' Missionary Union, Miss Miller, of 26 Horbury Crescent, W., sends us some interesting details of the work of the Union since its establishment six months ago. She also wishes to intimate that there is to be a general meeting for nurses from all the London hospitals from 7 to 9 on Monday next at the All Souls' Church Home, 54 Titchfield Street, W., where there will be addresses amongst others from medical missionaries, and where any nurse will be welcome. The Union now numbers 66 members, many from the London and provincial hospitals, others engaged in district and private work, and three in the mission field in West Africa. Miss Miller states that fully-trained nurses with religious convictions are much wanted. In Jerusalem a nurse is required for hospital work among Jews and another for work among Europeans. A third is needed in the spring to go to Shansi, North China. Nurses are wanted in Bangalore and Peshawar; two for China by the Church of England Zenana Missionary Society, and there are openings for more in the Church Missionary Society's hospitals at Quetta, Baluchistan, and at Pakhoi, South China. Miss Miller will be glad to give further details to any nurse who writes or calls to see her.

### NEW QUARTERS FOR HUDDERSFIELD INFIRMARY NURSES.

There has just been opened at Huddersfield a commodious building which has been converted from a school into a home for the nurses employed at Crosland Moor Workhouse. It contains nine separate bedrooms on the first floor, and on the ground floor there is provision for a general room, private sitting-room, and a large dining-room. The other half of the building is to be used as a sewing-room and clothing store, and there is also accommodation for a special class of old women, with a room for an officer who will have charge of this part of the block. After the formal ceremony, there was dancing by a number of visitors and the nursing staff.

### SHEFFIELD GUARDIANS AND THEIR SUPERINTENDENT NURSE.

The Sheffield Board of Guardians cannot be congratulated upon the way in which they have treated the recommendations of their hospital committee, who, in consequence of the increasing work devolving upon Mrs. Lawson, the superintendent nurse, recommended that her salary should be increased from £70 to £100. It was pointed out, in support of the proposal, that £100 is less by £22 10s. than the salaries paid in similar infirmaries in the six surrounding unions to the superintendent nurse, and no one denied the accuracy of the statement that Mrs. Lawson performs her duties in an indefatigable and efficient manner. Nevertheless, several guardians objected to the suggestion that she should be paid a salary which would compare a little better than £70 with that given by neighbouring boards, and the matter was referred back to the Hospital Committee for reconsideration. We hope that the latter will persist in the recommendation and resign in a body if it be not acted upon next time it is submitted. If their decision on such a point cannot be accepted by the guardians, the latter should be compelled to choose a committee in whom they have more confidence.

### SOCIAL GATHERING.

Another social gathering for Somerset nurses was held at Marycourt, Bridgwater, on November 20. Miss M. Foster Barham, who has undertaken to act as secretary for this centre, entertained the nurses most kindly. Several nursing appliances were exhibited, and the subject discussed was "Bed-sores, their Prevention and Treatment."

### Lectures Upon the Nursing of Mental Diseases.

By Robert Jones, M.D.Lond., B.S., F.R.C.S.Eng., M.R.C.P.Lond., Resident Physician and Superintendent of the London County Asylum, Claybury.

### LECTURE I.

For nursing the insane it is necessary to have sympathy, tact, and patience. It is further necessary to have a good and comprehensive knowledge of ordinary sick nursing. Before this can be satisfactorily attained, an acquaintance, more or less full, is expected with the structure and the functions—the anatomy and physiology—of the human body. The present course of lectures is in no way intended to supplant—but rather to supplement—the many excellent nursing hand-books on this subject, and it is presumed that those to whom they are addressed have received competent training in ordinary medical and surgical nursing, and having become efficient in this direction, are now proposing to qualify for mental nursing. The combination of hospital and asylum experience thus obtained is strongly advocated, and will do much to raise the status of the asylum nurse, as well as that of asylum nursing, but the time involved will, it is feared, prove a hindrance to many.

In well-organised hospitals and asylums for the insane, both private and public (county, borough, or district), mental nursing is taught to probationers by lectures, demonstrations, and ward or practical bedside work, involving a period of two or three years, in much the same manner that ordinary sick nursing is taught in hospitals.

Fig. 1.—*A*, cerebrum; *B*, cerebellum; *C*, pons Varolii; *D*, medulla oblongata; *E*, spinal cord. The names apply to the cerebral convolutions.

Fig. 4.—Spinal Cord. Side view plan of the Fissures and Columns.

1, Central canal; 2, white matter; 3, posterior median fissure; 4, grey matter; 5, anterior median fissure; 6, posterior root; 7, posterior ganglion; 8, spinal nerve; 9, anterior root.

Fig. 2.—Outside view of one half (left side) of a cerebral hemisphere, showing motor centres for the head, arms, and legs (*C*), the one for speech (*A*), and hearing (*B*).

Fig. 3.—Inside view of same half (left side) of cerebrum, showing motor area (*D*), as also those for sight (*A*), smell (*B*), and touch (*C*).

The study of mental diseases, although the highest—for it deals with mind, man's highest attribute—is yet the most difficult to understand, for there is often no bodily disease to treat, and no indication of a physical ailment. Still, the more minutely scientific investigation is carried out, the more changes are discovered in the minute structure of the

### LECTURES UPON THE NURSING OF MENTAL DISEASES—*Continued.*

cortex of the brain. It becomes advisable, therefore, and even necessary at the outset, that we should know something definite about the anatomy and physiology of the nervous system.

The group of organs which come under this system comprise (1) the *brain*, (2) the *spinal cord*, (3) the *nerves* and their terminations, and (4) the *sympathetic system*—so called because some of the fibres from the sympathetic system supply the thoracic and abdominal organs, those going to the heart exciting it to beat faster; whilst those supplying the intestines moderating their contraction, and those to the blood-vessels maintaining their tone.

The brain within the skull cavity—the vault of which protects it from injury—is enclosed in three membranes, called the *meninges*, which may become the seat of a fatal inflammatory disease called *meningitis*. The outermost membrane, the *dura mater*, is tough and fibrous, and forms a lining membrane to the bone. The innermost, the *pia mater*, forms a supporting membrane to the brain, dipping into all the inequalities of its convolutions and supplying it with blood; whereas the middle membrane, the *arachnoid*, more or less lines the other two, passing over the convolutions and not dipping in between them. It thus forms a kind of bag or cavity. This cavity contains a fluid, the cerebro-spinal fluid, which acts as a soft cushion, moistening the surface of this cavity, preventing concussion of the brain, and to some extent regulating the amount of blood within the brain. For the spinal cord the cerebro-spinal fluid acts as a water-bed to prevent jars or blows.

The brain (fig. 1) is divided into the (A) *cerebrum* or great brain, which includes by far the largest part of the brain of man, and which comprises two equal halves, the cerebral hemispheres, the (B) *cerebellum* or little brain, which is in less well-marked halves, joined in front by the (C) *pons*, a bridge of nerve fibres. The brain continues into the *spinal cord* by means of a stem called the (D) *medulla oblongata*. From the spinal cord nerves—31 pairs—go all over the body.

The most important part of the cerebrum is the outside grey matter, or cortex or rind. It contains millions of nerve cells, each of which is provided with many interlacing processes called dendrons, and with one nerve fibre, the axis cylinder which conveys nervous impulses, and which is the essential part of a nerve fibre—like an electric bell wire.

The cortex of each cerebrum (fig. 1), which is divided into four lobes—(a) the *frontal*, (b) *parietal*, (c) *occipital*, and (d) *temporo-sphenoidal*—is crinkled into folds or convolutions, as seen in the figure, and the greater the brain power of a man the more convoluted, it is surmised, is his brain cortex.

The cortex is the seat of motor areæ, as shown on figs. 2 and 3, where the centres are marked for the head, arms, lower limbs, and the trunk. These areæ or centres have been successfully mapped out in the monkey.

The cortex is also, probably, the seat of consciousness and of the different sensations (figs. 2 and 3), *i.e.* auditory or hearing, visual or sight, olfactory or smell, tactile or touch, which sensations arrive from the surface of the body. The cortex both receives and gives out nervous impulses. Those travelling to the brain (such as, for instance, from the special sense organs, the eye, ear, etc.) are called *afferent* impulses, which, as a consequence of changes set up in the nervous substance of the brain, proceed from thence to the muscles, glands, and other organs, as *efferent* or outgoing impulses.

Nerves carrying sensation to the brain are also called sensory nerves as well as afferent, and those carrying outward impulses—efferent nerves—are also called motor, because their principal effect is to produce movement. When afferent impulses fail to give rise to sensation—such as when a child's foot is tickled during sleep, and it does not wake, but the foot moves responsively, the resulting movement is called a *reflex* action. Swallowing is a reflex action, the food stimulates the back of the throat and is swallowed without conscious effort. Walking may become a reflex action, when two persons walk together engrossed in conversation and unconscious of their paces. The action of the pupil of the eye is reflex, and highly-acquired complex movements, such as when a person plays the piano and is at the same time more or less engrossed in conversation, may, for the time, be carried out reflexly and without rousing consciousness.

The *sympathetic system* consists in small masses of nervous structure called *ganglia*, mostly on either side of each vertebra in the thorax and abdomen. These ganglia are connected with the other nerves, motor and sensory, already referred to. It is important to remember that efferent nerves from these ganglia proceed to the different organs in the chest and abdomen, and other nerves from the organs carry impulses to the ganglia and are afferent in their nature.

The average weight of the brain is about 48 ozs. (1,360 grammes) for men, and 43½ ozs. (1,230 grammes) for women, although some eminent men have had much heavier brains. The brain grows very quickly until the fifth year, then very slowly. After twenty growth is not perceptible. In proportion to the size of the body, man's brain is pre-eminent in complexity and weight. A whale, 70 feet long, has a brain weighing only 80 ozs., and an elephant's brain is only about 128 ozs.—only three times as large as a man's.

*The Spinal Cord* (fig. 4) is that part of the nervous system which lies in the spinal canal. It is thicker in the cervical (neck) and lumbar (loin) regions than in the dorsal, because in the thicker portions the great nerves to the arms and legs come out of the cord and swell it. The cord itself ends in a fine extremity, called the *filum terminale*, but at the lower end the pairs of nerves already referred to as coming out between each vertebra travel down some distance in the spinal canal, forming a bundle of nerve fibres called the *cauda equina*, or horse-tail, owing to its appearance.

There is a slit, or fissure, in the middle line of the cord in front, all the way down, and another at the back, called respectively (fig. 4) the (5) *anterior* and (3) *posterior* median fissure. At the bottom of each fissure is a bridge of nervous tissue surrounding the (1) *central canal.*

Like the brain the spinal cord consists of grey and white matter, but—unlike the brain—the grey matter in the cord is inside, and the white matter outside. The latter is mostly nerve fibres, whereas the grey matter consists of nerve cells, as well as fine fibres. The section, fig. 4, shows the two crescents of grey matter, back to back (4).

Each spinal nerve is connected with the cord by two roots, the (9) *anterior*, or motor, and the (6) *posterior* or sensory root. Just before they unite the posterior is enlarged into a swelling called the (7) *posterior root ganglion.* The (6) posterior root conveys sensory impulses to the cord and brain (afferent), and the (9) anterior conveys impulses (motor) from the cord to the muscles (efferent). Each spinal nerve is thus composed of both kinds of nerve fibres.

Besides *sensory* and *motor* fibres there are also *secretory* nerves to the various glands and *trophic* nerves, which control the growth and well-being of the parts of the body to which they are distributed.

This much seems necessary before we commence with the functions of the brain, and the disturbance of these functions such as occurs in disease.

### Incidents in the Bombay Fever Wards.

BY A NURSE IN INDIA.

OUR patients are of many nationalities and diverse creeds, but of course Hindus largely predominate, and these, if we except a considerable number of beggars and roadside cases, come chiefly from the labouring or coolie class, and are not, as a rule, natives of Bombay, but flock in from Rutnagiri, Satara, Poona, and other distant places in order to obtain work at the mills or about the docks. Elderly men, anxious to add to their scanty income, leave wives and children on their little plot of land in the far-away gaum (village) and work here for the greater part of the year. Sometimes the women accompany them, husbands bringing with them the eldest boy or girl, should he or she be old enough to go to work and help in the housekeeping, or perhaps they bring the little babe still too dependent upon its mother to be left to the care of a Suggá Wallá (relation), as the older children are. They have a deeply-rooted love for their native place, and weary to return to it when ill. In hospital many pathetic scenes are the results of this separation of families.

### In the List of the Dead.

Occasionally a grey-haired woman arrives from up-country and beseeches us to tell her where her son is. We must know him, he is a very strong man. A Gâum-Walâ (neighbour) has written to tell her of his illness, and that he had been taken to our hospital. With a strange sense of misgiving we bid her look round the wards and see if she can identify him. No, he is not here! Where is he? Has he got well and gone away? He was a very strong man. There is another alternative we know, and our misgiving grows stronger as we take her to the office and ask the clerk to look up the books and see whether any discharged patient is entered under the name she gives. Ah! The name is soon found, but alas! not among the list of discharged cured. The young man's name is among the list of the dead! The old woman will not at first believe it; it must all be told her over again. Then she covers her face with her *lugádá*, and with a heart-broken cry of "Átá mé Káyá Karu!" (now what shall I do) totters away. Or it may be that no such name is to be found. "He has not come here," we say; "you had better inquire at the Maratha Hospital." "Oh she has been there already and not found him; the people there advised her to come to us." Then to Modi Khana. Have you been to Modi Khana? She has never heard of Modi Khana. Where is it? she asks. Modi Khana is at least four miles off, but the tramcar will take her most of the way. We advise her to ride. She has money for the fare, and she starts off once more on her sorrowful search.

### A Contrast.

Although scenes of a terribly tragic and sadly pathetic character are constantly taking place in our wards, the work is not devoid of a strong element of humour. The look and gesture of the people as they beg for the forbidden *bhakri* (native bread), and measure the amount they plead for on their fingers; the folded hands, the up-raised eyes, the forehead bent low till it touches the bed as the patient informs the doctor, *Malä ghari jayälä pâlije!* (I want to go home) are quaint to a degree. So also is the expression of surprised innocence which the patient puts on when caught secreting unwholesome food, brought him in cruel kindness by some friend, and surely it is hardly possible to express a smile, when as we go to take a man's temperature he suddenly pops his tongue out for inspection, or in answer to your question, *aja kaissa ahai* (to-day how are you) he mutely offers his hand that you may find out yourself from his pulse. It is comically embarrassing, too, to have one's feet unexpectediy embraced by a grateful pair of arms, especially if one happens to be walking hurriedly across the ward with hands fully occupied holding milk, medicine, or a baby! Four o'clock in the morning is, perhaps, the most amusing time. Then the ayahs and ward-boys begin to change the sheets and make the beds, and the convalescent patients get up and help them. They looked very odd during the cold weather squatted round the ziggaries (small iron pans for charcoal) trying to warm themselves. One would fancy that they would be better off in their comfortable beds at that early hour; but no, the ziggaries have an irresistible attraction. Others perched upon the cots with knees drawn up to their chins and so wrapped up in blankets that only their faces are visible, extract from unsuspected hiding places their much-loved *biddees* (cigarettes), and prepare to enjoy a morning smoke. They are very generous in sharing these luxuries with each other, and one may often see two men puffing contentedly away, turn and turn about, at the same biddee.

### Hindu Children.

While the men smoke, the children, also enveloped in blankets, till all that is seen of them is the gleam of their bright eyes and ivory-like teeth, watch us anxiously to see if any of the ever-welcome "*biskuts*" are coming their way. Hindu children are delightful, especially the little ones. Their favourite head-gear is a curiously-shaped bonnet, made usually in two colours of silk or chintz. The crown is quite square and sits right on the top of the head, whilst a straight piece goes across the forehead. The crown is decorated in the centre and at each of the four corners with a tassel, which stands up straight, and gives to brown baby-faces beneath it a very droll appearance. Nothing pleases a Hindu woman more than dressing her child in one of these caps. She seems to take it as a personal compliment, and from the time the little sable head is packed up in one, the mother appears to be convinced of one's kindly intentions.

### A Pathetic Story.

The story of seven-year-old Ballia is a pathetic one. He and his father were victims of the dreadful famine and must have undergone great privations before they drifted into hospital, suffering from the terrible relapsing (famine) fever. The poor father was too far gone for recovery to be possible, and he soon died. Ballia, who lay on the next cot, was asleep at the time, but when he awoke to find his father's bed empty and was told the reason, he was overcome by grief, to which he gave vent in shrill and continued screams, nor could anyone quiet him. At last in despair we offered him two pice, that unfailing panacea for the woes of a Hindu child. He grabbed at the coppers and the screaming stopped. We were thankful, but it seemed a callous way to comfort an orphan. Presently, however, the child called me to him saying, in a stifled voice, as he pushed the money towards me, "I don't want pice, I want my father," and the terrible screaming began again. The people of India are almost childish in their fondness for children, and everybody was good and kind to Ballia. The ward-boys were only too ready to spoil him, whilst one of the convalescents took him under his special protection. For two or three days the child seemed happier; then, alas, his friend was discharged. That night Ballia woke up and found no one at hand to comfort him. The sense of his sorrow overcame him afresh, and he fell into a violent paroxysm of grief. "Give me a knife," he cried, "that I

may kill myself. I want my father, I want to go to my father! Let me die." Poor bairn! what sights and sounds his baby eyes and ears must have heard and seen, for him to think of such a thing at his tender age!

### Old Bhimabai.

Bhimabai's story was to her a tragedy, but it had a somewhat comic ending as far as the hospital staff was concerned. She was a respectable old woman, a fruit seller, tall and rather good looking, with straight, well-shaped limbs, a wonderful head of hair, thick, wavy, and perfectly white. She had been found unconscious in the compound of a house on Malabar Hill (whither she had probably gone to sell her wares) and sent into Arthur Road Hospital. Her ailment was speedily diagnosed recurrent fever, and her temperature fell in a few hours from over 107 degrees to many degrees below normal. During the second night after her admission she suddenly awoke to consciousness and began to cry out unceasingly that she had been robbed, that her jewels and money were gone. She refused all food and medicine, and not only would not sleep herself, but disturbed the other patients. Now it frequently happens that our relapsing fever patients are wildly delirious when their temperature is sub-normal, and having ascertained from the clerks in the office that Bhima had no property on her person at the time of her admission, we fancied that her raving was due to delirium, and did our best to quiet her. Dhondoo, one of the ward boys, who was always very kind to the patients, sat down by her bed and told her, as he patted her cheek and soothingly stroked her snowy tresses (regardless of the laughter of his fellow-workers and the convalescent patients) that he had the jewels and money quite safe at home and would bring them to her on the morrow. Day by day Bhima grew more frantic, and would entreat with tears and most pathetic gestures everyone who came near her to restore her lost property; and at last the doctor, wearied with her importunity, discharged her.

No sooner did the old body leave the hospital than off she went to the nearest police station, and presently returned with an European Inspector, before whom she charged Dhondoo and another ward-boy with having stolen her money and jewels. In vain they protested their innocence. They had to submit to having their quarters searched, and might have been marched off to jail had not the Doctor opportunely arrived and explained matters, referring the Inspector for further information to the officer who had found the woman and sent her to us. We never heard how the matter ended, but Bhimabai's story was perfectly true. She had been known to the police as a fruit-seller for twenty years. Many of her neighbours testified to her having always worn a handsome silver belt, besides bangles and other jewels, the savings of a life-time. It was proved, too, that her brother-in-law had, the day after her admission into hospital, given information of her disappearance to the police, and had stated that she had left her house wearing these ornaments. Probably when overcome by the fever she had sunk down far from home, and some unprincipled person had discovered and robbed her.

### Presentations.

Warrington Isolation Hospital.—The Matron of Warrington Isolation Hospital, Miss Cameron, who is resigning, has been presented with a silver cream jug and sugar basin, of Jacobean period, "as a mark of affection from the nursing staff." The domestic and outdoor staff gave her a copper and brass kettle on stand, also a glass salad bowl and servers.

### New Books on Nursing.

The Care of Infants: A Manual for Mothers and Nurses. By Sophia Jex-Blake, M.D. Second Edition. (Edinburgh: G. A. Morton. 1903. Pp. ix. 109. Price 1s. net.)

This is a reprint, with three short footnotes newly added, of a useful little domestic manual published 18 years ago. As it consists mostly of practical common sense, it is as true to-day as when it was written, while the physiology in it is of so rudimentary a kind that it does not get out of date. Dr. Sophia Jex-Blake believes that the great majority of the deaths of children under five "could be easily avoided by common care, combined with the most elementary knowledge of an infant's nature and needs." Ignorance and neglect, she alleges, are the wholly preventable causes of the enormous waste of life in infancy. Her object is prevention; and as she is not concerned with the moral, social and economic causes of infantile mortality, she devotes herself with all the greater zeal to those immediate practical matters which are within the competence of every mother and nurse. Her directions upon feeding, clothing, nursing, bathing, etc., are comprehensive and minute, and are pointed against the mistakes arising from ignorance or erroneous traditions, which her observation has shown to be most frequent.

The Guide to Household Nursing. By Miss J. A. Andrew. (Published by the Walter Scott Publishing Company, Limited, London. Price 6d.)

This is an unpretentious little book for which we predict a sphere of much usefulness. It is compiled by the superintendent of the Gateshead Nursing Association with the intention of setting forth in simple language the essential principles of order and cleanliness in a sick-room together with a certain amount of elementary nursing technique. The volume is intended for those who, without special knowledge, are "attending to the sick in their own homes," and have not the assistance of a trained nurse. Miss Andrew never loses sight of the character of her readers nor does she forget how very limited may be their resources—her directions are always clear, and the most ignorant cannot fail to understand them. It is a distinctly happy thought to suggest that when there are a number of helpers one always should be responsible for the medicine and dietary, and that if possible the same person should not undertake both day and night work. The chapter on "The Bedroom" is remarkably good, but in the section on "Ventilation" more stress might be laid upon the necessity for open windows, even though the chimney be unstopped. The fear of fresh air is always a difficulty in cottage nursing, and the inch of open window advocated by Miss Andrew is far too low a standard. The paragraphs on "Bed-sores," their prevention and treatment, are particularly good and practical, and the directions as to the care of the patient's mouth are very clear and sensible. Bearing in mind the extreme ignorance of the poor, it might be well in a future edition to mention that in cleansing eyes a fresh piece of rag should be used for each eye, and we fancy that it is better to fill a water pillow after, rather than before, it has been placed in position. The need of great cleanliness for babies' bottles and milk vessels, and a simple method of sterilizing milk, might be fully explained; and surely it is a mistake to advise even presumably healthy persons to swallow their expectoration, as distinguished from saliva. The presence of tubercular disease may not have been detected, in which case there might be grave risk of setting up intestinal mischief. Scattered throughout the book are many most useful hints, and the details (especially as regards the giving of medicines, hot applications, the storage of ice, and household cleanliness) will, we feel sure, be of the utmost help and value to many a poor woman.

### Everybody's Opinion.

### MALE NURSES.

"Trained Male Nurse" writes: In The Hospital for the 14th inst. you state, in reply to "A. C. T.," that the National Hospital, Queen's Square, is the only hospital in Great Britain where male nurses are trained. Permit me, as a male nurse with over 16 years' experience, to correct you. By far the greater number of private male nurses have received their training in the Royal Army Medical Corps. I myself, after upwards of five years in military hospitals, took up private nursing, and am still in harness and very successful—my fees during the 11 years rarely averaging less than 150 guineas. I think I may claim that, as in the Royal Army Medical Corps, the training is a more general one than in the National Hospital, so other things being equal, the best male nurses are turned out thence.

[The answer in question only had reference to civil hospitals.—Editor Hospital.]

### ASEPTIC FLOOR FOR OPERATING THEATRE.

"Surveyor" writes: In reply to "S. L.," after some years' experience in hospital work, I have come to the conclusion that asphalt or mosaic are the two best flooring materials for theatres. The only objection to asphalt is its colour, *i.e.* black, but for cleanliness there is nothing to equal it. Mosaic makes a good hard wearing floor, and if well laid there should be no open joints to become septic. The cause of cracking in mosaic floors is often the excessive size of steel joists used in the construction of the floor. Having had a large experience of hospital work, and to help a small hospital, I will, if agreeable to "S. L.," inspect floor and give my report on same, free of cost, if hospital is in London or suburbs, or for out-of-pocket expenses if away from London. I enclose my address.

### POOR LAW SUPERINTENDENT NURSES.

"An Irish Superintendent Nurse" writes: While the fate of Poor-law nursing in England hangs in the balance may I say that we in Ireland have for some years past been independent of the master and matron and subject only to the medical officer? I need hardly say that in spite of this wise and advanced policy of the Irish Local Government Board continual friction will arise in those small unions where the infirmary does not possess its own laundry, adequate paid staff, etc., unless the nurse and matron are friendly and considerate to one another. The high pay (£40 or £50 and frequently money in lieu of rations) given to secure a trained charge nurse—and no other will be sanctioned by the Local Government Board—tempts many a woman who is profoundly ignorant of domestic economy to undertake the work, and thus by waste and extravagance she arouses the contempt of the matron who has, perhaps, for many years conscientiously looked after the pounds, shillings, and pence of the ratepayers. Not more than one case in ten requires any real nursing; half the patients may be up and dressed every day, but all need careful washing and feeding, and even with the monotonous diet and few "extras" a nurse with knowledge of cooking can often tempt a failing appetite, and by a little planning and encouragement keep all the linen mended and darned by the patients. My experience is that nurses often think it *infra dig.* to sew on a button, and I know that in a general hospital time could hardly be found to do it, but in little country infirmaries a nurse should be ready to turn her hand to anything. Even gardening may be a delight not only to herself but of immense interest to the patients, some of the men being always ready to help, and in this way she becomes a real influence for good and a valued servant to the community. The constant excitement of interesting cases and the consequent wear and tear of ordinary hospital life are wanting, and the pauper patients are largely of a very low class and great firmness and patience are needed in dealing with them. For these reasons I think the Poor-law service eminently adapted to those older nurses—I myself am 47—who want rest after years of "sturm and drang," with a position of comparative independence and a pension to look forward to in their old age.

### POOR LAW VACANCIES AND GENERAL HOSPITAL-TRAINED NURSES.

"Jean" writes: I also am a Poor-law nurse and an interested reader of the correspondence in The Hospital about hospital-trained nurses and workhouse infirmary appointments. We should like to hear what hospital nurses have to say for themselves. I suppose it is the better pay that attracts them into the Poor-law service. I hope infirmary nurses will not allow the subject to drop until something has been done. Could not you, Mr. Editor, advise us what we ought to do? We should be very gratified if you would, as we have great confidence in your judgment. All the sisters and nurses in this large infirmary agree with "Margaret," and would gladly do anything in their power to stop this "invasion."

### WORKHOUSE NURSING.

"Dr. J. T. Macnamara, Medical Officer, Ladywell Workhouse," writes: In your otherwise correct summary of my few remarks at the meeting of the Hospitals Association, held in the Governors' Hall of St. Thomas's Hospital, one error occurs which I trust you will allow me to correct. I am made to say that "when the matron and superintendent retired there was no nurse in charge at all." What I believe I said, at any rate what I intended to say, was that "when the assistant-matron, who happens to be a trained nurse, and the superintendent nurse retired to rest there was practically no nurse in charge at all. You will, I am sure, see the importance of this correction as the present assistant matron had been for a considerable time superintendent nurse at Ladywell and undertook the duty when recently there was supposed to have been "friction" at the institution.

### NURSES AS STEWARDESSES.

"Nautical" writes: I feel it my duty to write and contradict the statement "W." made in The Hospital a week or two ago about "Nurses as Stewardesses." I am a trained nurse, and have also been a stewardess. I am the daughter of a naval medical officer. Being left in reduced circumstances, my three sisters and self were obliged to turn out into the world. Two of my sisters went to sea as stewardesses in the P. and O. Company, and my younger sister and self have trained as nurses in London hospitals. I have since been a few trips abroad, so I know exactly what the work of both nurses and stewardesses consists of, and I must say that a stewardess in the first saloon of a good liner has not half the menial work a hospital nurse has to do. I do not understand what "W." means about sweeping, beating curtains, washing up cups and saucers, etc., etc. This plainly shows the very inferior class of ship she must have been on, perhaps one of the "excursion barges" which run up the river Thames at cheap rates during the summer months. I have crossed over to Ireland several times as a passenger, and even on those boats, which are, of course, not to be compared with the large liners, the stewardesses have told me what an easy time they have and what nice work it is. "W." speaks of the "cabin boy"; I suppose she means "steward." I should hardly think there would be only one, however small and second-rate the ship might be. I have always found stewards most willing to do anything for me, and most polite in every way. As far as money presents are concerned, I have never felt in the least awkward nor humbled in any way; in fact, I have always found my passengers exceedingly nice and generous.

### THE USE OF COLLODION.

"A Nurse" writes: I was sorry to see that the examiner of the replier to the examination questions condemns the use of collodion as a "pernicious thing" in the prevention of bed-sores. From practical experience I have found it act splendidly as a protection to the skin, and as a means of keeping a dressing on a bed-sore in place I think it has no equal. A short time ago I was working for the Queen Victoria Jubilee Institute. In my district I had a case of paraplegia. When I first saw the patient, a man 23 years of age, he had a large bed-sore on each of his hips, yet was always obliged to lie on his side, as he experienced great pain when on his back. I attended him for many weeks and dressed his bed-sores twice a day with zinc ointment spread on lint. I also obtained a water-bed for him. After some time I was getting into despair about the healing of those bed-sores, as I could only pay him two visits daily, and, with all the attendant discomforts of paraplegia to contend with, I found it was almost impossible to keep a dressing either dry or in position. At last I determined to try collodion. I then dressed the sores as usual with zinc ointment spread on lint; over this I laid a single layer of cyanide gauze, cut about half an inch larger all round than the piece of lint, and I painted the whole and the surrounding skin with collodion. I dressed both bed-sores in this way twice daily, each time removing the shiny surface of the collodion and well washing all round. The result was that the dressings were never in the least out of place and were kept dry, both bed-sores quickly healed, and the surrounding skin was protected. In the same year I nursed a private patient, an elderly lady who was extremely thin. This was a case of thrombosis and paralysis, and the doctor had said that bed-sores were almost inevitable. I used for her zinc ointment and sometimes the white of an egg and whisky, but in spite of every care I noticed one morning when washing her that in one part the skin looked very tender and wrinkled and almost on the verge of breaking. After carefully washing with ordinary yellow soap I painted over the tender-looking part with collodion and continued to use it every day for any specially tender places, with the result that, though the patient lingered for many weeks, her skin was kept perfectly intact. I have ever since regarded collodion as a nurse's best friend in the prevention of bed-sores.

### NURSING CHOREA.

"Sister Ellen" writes: In my rounds of the medical wards I have frequently had the same answer from nurses in response to inquiries for new cases admitted, "Only a case of chorea." To many nurses such cases present small attraction and little interest, but no cases test a nurse's skill and patience more. The majority of patients are young and somewhat frightened at their own jerky movement, so that a nurse's first aim should be to gain the child's confidence and to assure the patient that he is not being laughed at. Many patients will refuse food rather than attempt to feed themselves; therefore much patience is required to help the invalid to take food slowly. If in a large ward, it is desirable to screen the patient during meal times, also to give the food ordered in small quantities, as in small children they might swallow it without masticating. Each medical man has his own treatment, and if arsenic is prescribed, which is frequently done, the nurse must watch for vomiting or any symptoms of too great susceptibility to the drug, at once reporting to the physician. The patient must be kept at rest, free from excitement, and in fits of crying or laughing which are frequently quite uncontrollable, sympathy, but firmness, must be shown. The patient's heels and elbows must be carefully examined for sores, as the frequent movements soon develop such in severe cases. It is a wise precaution to wrap the part in wool at first. After some years of observation I have noticed that the increase of cases is usually about the time of school examinations, when a nervous child is being overstrained, and in large towns underfed, with the result of a breakdown in the nervous system. If an extra meal could be provided at the Board Schools during times of extra pressure, children would be helped to pass better, and in many cases probably be saved this distressing illness. If the malady has been induced by overstudy, all books must be forbidden. If the case recovers quickly the nurse may take credit to herself, as more can be done by skilled nursing than by drugs. She will also have the satisfaction of seeing her patient at rest, in contrast to the contortions and violent movements when admitted.

### Appointments.

Barton Regis Union Hospital.—Miss Eva Gertrude Monks has been appointed superintendent nurse. She was trained at the Bristol Workhouse Infirmary, Stapleton, and afterwards became staff nurse. Since then she has been charge nurse at Barton Regis Union Hospital. She holds the L.O.S. certificate.

Birkenhead Borough Hospital.—Miss Kate Theodosia Beatty has been appointed matron. She was trained at the David Lewis Northern Hospital, Liverpool, and has since been temporary ward sister in the medical and surgical wards, sister-housekeeper and home sister at the same institution.

County Hospital, Newport, Mon.—Miss M. E. Barr has been appointed staff nurse. She was trained at Hull Royal Infirmary, and has since been attached to the private nursing staff.

Hungerford Workhouse Infirmary.—Miss Emma H. Hawkridge has been appointed superintendent nurse. She was trained at the West Ham Infirmary. She holds the L.O.S. certificate.

Southwark Union Infirmary, East Dulwich.—Miss Agnes Corrie has been appointed sister. She was trained at the North Staffordshire Infirmary and Eye Hospital, and has since been charge nurse at the Eastern Fever Hospital, London, Queen's nurse, and private nurse. She holds the L.O.S. certificate.

Western Infirmary, Glasgow. — Miss Clementina Kemp has been appointed night sister. She was trained at the Western Infirmary, Glasgow, has been charge nurse at the Fountain Fever Hospital, London; she also served for two years in South Africa as a member of the Army Nursing Service Reserve, and since her return she has been attached to the Scottish Association of Trained Nurses, Alva Street, Edinburgh.

### Novelties for Nurses.

By Our Shopping Correspondent.

### A NEW NURSES' PINCUSHION.

Nurse Ellison sends me a pretty star-shaped nurses companion, which she calls "The Star of Hope." It is made of scarlet satin in the front, which is intended for safety pins. Round the edges pins, large and small, are stuck in. The reverse side is flat, covered with red flannel, for threaded needles. The thimble hangs in a ring of red wool in front, just beneath a corresponding ring which is intended for attachment to the chatelaine. The position of the thimble does not seem very secure, and is the least practical arrangement connected with the pincushion. "The Star of Hope" may be obtained from Miss F. Johnson, Hazeldean, Cargate Avenue, Aldershot. The price is 1s. 6d.

### TRAVEL NOTES AND QUERIES.

By our Travel Correspondent.

Rome by Different Routes (E.C.)—Second-class fare single is £7 9s. 11d. *via* St. Gothard and Bologna; *via* Mont Cenis, £6 16s. 9d.; *via* Dieppe, Turin and Pisa, £6 2s. 10d. Going by sea from Genoa, it works out like this: *via* Dieppe, Paris and Turin, to Genoa, £4 7s. 2d. Thence by steamer to Leghorn, and thence by rail to Rome—about another £4 10s. You see it is considerably dearer to divide the journey in that way. The cheapest route *via* Dieppe, Turin and Genoa is very comfortable. I generally go that way. If you go direct to Naples the cheapest route is the same, route as above, *via* Dieppe and through Rome, £7 0s. 7d. The Orient line has steamers running from Tilbury straight to Naples fare £11. I have not been able to learn particulars as yet of the local steamers from Genoa to Naples, but I will insert information when I have obtained it.

### Echoes from the Outside World.

### Movements of Royalty.

King Edward and Queen Alexandra took the opportunity, of the absence of their Royal guests at the Guildhall last Thursday, to visit the Irish Industries Association's Sale of Work, which was held at the White Hart Hotel, Windsor. The stalls were presided over by Princess Christian, the Duchess of Argyll, Lady Dudley, Lady Cadogan, and others. The King bought quite a number of Irish knitted socks for his own personal use, as well as some tweed for a suit. At almost every stall he made some purchase, and at last he had to laughingly refuse to become the owner of anything more, because he said that he had spent all his money. The Queen also made a selection of many articles for herself and her grandchildren, and for charitable purposes. But their Majesties were prevented from attending the second day. Following on a Sunday spent quietly at Windsor, the Royal Family, including the Duchess of Fife and Princess Charles of Denmark, and accompanied by the Grand Duke and Grand Duchess of Vladimir of Russia, returned to town on Monday morning. After luncheon at Buckingham Palace their Majesties, with Princess Victoria and Princess Charles of Denmark, went on to Sandringham, where they will remain for the present, the King going on a visit till the end of the week to Lord Farquhar, at Castle Rising.

### Departure of the King and Queen of Italy.

The only public function which the King and Queen of Italy attended during their stay in England was a banquet at the Guildhall given by the Lord Mayor on Thursday. The weather was crisp and bright, and thousands of people assembled all along the route from Paddington Station to the City. The reception given to the Royal visitors was most enthusiastic, and they appeared immensely pleased. Her Majesty wore a dress of pale grey silk, over which was a long cream plush coat trimmed handsomely with sable and finished by a deep cape of guipure lace. The King was wearing his uniform as a general of the Italian army. Three addresses were presented, at the Embassy, the Westminster Boundary, and Oxford Circus. The Duke of Connaught and his daughter, the Prince and Princess of Wales, and Signor Marconi were amongst the guests who arrived just before their Majesties, and all were conducted to the library where an address in a gold casket was presented. The King, who is a famous numismatist, was the recipient of a complete set of medals struck by the Corporation to commemorate great occasions. Later, when the guests adjourned to the Great Hall, there was an elaborate luncheon for 840 visitors, the service for the Royal visitors being all of gold. The King of Italy responded to the toast of his health in excellent English. On Friday the Royal sportsmen shot in the Great Park all day, the two Queens visiting the mausoleum at Frogmore and afterwards joining their husbands at luncheon. A concert took place in the evening, and on Saturday morning the King and Queen of Italy returned home. The leave-taking of their host and hostess was most cordial, and the Prince of Wales went as far as Portsmouth to see the Royal visitors on board the Royal yacht.

### Death of Three Members of Parliament.

This week the death of three well-known members of the House of Commons has been recorded. Lewisham has been deprived of the services of Mr. John Penn, who was first returned for the borough in 1891, and who, though he rarely spoke in Parliament, was as first rate a man of business as he was good a golfer. Mr. Seale-Hayne, whose death creates a vacancy for the Ashburton division of Devonshire, which he had represented since 1885, filled the office of Paymaster-General when the Liberals were last in power. He was also a Privy Councillor, and though a very wealthy man, an extremely hard worker. When the South Devon Militia was embodied during the Crimean War and the Indian Mutiny, he served with it at Plymouth and Waterford, and was for many years musketry instructor to the regiment. Sir John Blundell Maple, whose death, like that of Mr. Penn, followed a long illness, had sat for Dulwich since 1887. He was the head of "Maples" in Tottenham Court Road, and was also a keen and highly successful man of business. He gave away a great deal in charities, and presented the city of St. Albans with a large park and recreation ground.

### The Guild of Handicraft.

The second annual Exhibition of the Guild of Handicraft is now being held at the new gallery of the Guild in Bond Street, and will remain open till Christmas. There is much that is beautiful and artistic, but the special feature of interest to the general public is the splendid volume which has taken the Guild about two years to finish, and which, by express permission, is called "The Prayer Book of King Edward VII." The issue is limited to 10 copies on vellum—of which one is the presentation copy to his Majesty—and 400 copies on paper. The work is profusely illustrated, a portrait of the King appearing at the beginning and a small one in a circle wherever the King's prayer is used, the last decoration in the new order for Accession being a picture of Queen Victoria. There are views of London, portraits of persons of distinction associated with the history of Christendom and the Church in this country, and decorations for the principal chants and the Holy Communion service. Altogether there are 150 woodcut block illustrations. Besides this book there are specimens of handicraft in metals, enamels, etc., book printing, illuminated writing, and binding done by members of the Guild.

### The Late Mr. Seton Merriman.

The sudden death from appendicitis of the popular author who wrote under the *nom de plume* of Henry Seton Merriman is announced. The real name of the novelist was H. Stowell Scott. Among his best known books are "The Sowers," "In Kedar's Tents," "With Edged Tools," "The Slave of the Lamp," and "The Vultures." The last was "Barlasch of the Guards." He was still a comparatively young man, and it was hoped would further distinguish himself in the world of letters. All his stories are marked by intellectual force, and they are told in polished, and often vivid, language. Mr. Seton Merriman was always more or less of an invalid, and he was exceedingly retiring. He steadfastly refused to be interviewed, and disliked talking about his own affairs.

### The Society of Portrait Painters.

The Exhibition of Pictures, which has recently been opened at the New Gallery, differs from most of the other London exhibitions, in so far that some works are old friends, others are new exhibits, and others, again, are loans from foreign artists of distinction. The Society of Portrait Painters this year pays a tribute to one of its original members who has lately passed away from our midst, and hangs a laurel wreath over the unfinished full-length portrait of a lady, by Mr. Whistler, which is entitled "Rouge et Noir, l'Eventail." The collection is rich in Orchardson's works, and includes, besides "Sir David Stewart" in his robes as Lord Provost of Glasgow, a portrait of an elderly lady, which is almost new to the general public. One of the most remarkable pictures is a group by Señor Zuloaga, the Spanish artist so much sought after just now in the French capital, and altogether admirable is "Madame Besnard," painted by her husband. Of modern British artists it is well to look out for Mr. Collier's "Mr. Richard Gunstone," Mr. Brough's portrait group of "Mrs. Willie James and Her Daughters," and the "Miss Goldie," of Mr. Alfred Hayward.

### For Reading to the Sick.

ADVENT—"BE YE ALSO READY."

NOT heralded by fire and storm,
In shadowy outline dimly seen,
Comes through the gloom a glorious form,
The once-despised Nazarene.
"Fear not, beloved, thou art Mine,
For I have given My life for thee,
By name I call thee, rise and shine,
Be praise and glory unto Me!
Thy life is hid in God with Me,
I stoop to dwell within thy breast."
"My joy for ever Thou shalt be,
And in my love for Thee I rest!"

*F. R. Havergal.*

Unto you is given
To watch for the coming of His feet
Who is the Glory of our blessed Heaven;
The work and watching will be very sweet
Even in an earthly home,
And in such an hour as you think not
He will come. *B. M.*

The hour is coming when each of us—with a consciousness of soul as clear as the sight of the eye of the body—when each of us shall see the fairest, the most awful vision, the coming of Christ! Here we see but dimly; *there* will be the full revelation.

. . . When Christ comes, this is certain, He will come revealing "hidden things of darkness," ay! and hidden things of light. It will be a time of unveiling. . . . It will be a moment of startling and complete revelation.

Revealed. Yes, what we want is that revealing. It is coming through the forces won and stored in the Passion. Christ crucified—revealing God, judging sin, perfecting holiness—is coming; the Light of the world is dawning; "every eye shall see Him." "There shall be no night there."

Let us look to that day with a sense of awe, indeed, deep and serious; but let us also look onward to that day with a prayer for increased and increasing desire that we may have grace to "love His appearing." Very gradually He trains us, but, if we allow Him, train us He does.

Life here is in deepest shadow, but nothing, since the beginning of creation, has been so wrapped in shadow as the fact and the consequences of Calvary; if that be clear, all must be plain. And clear it will be. And with it all those inscrutable mysteries so closely related to the Passion of the Redeemer which crowd around our passing footsteps as we march across the isthmus of life. These will be seen unveiled, unclouded in the full vision of the Coming Christ. Christ is the Great Revealer, in Him we shall see all.

We are weak and helpless, but He is mighty; our hearts are failing, but He is strong, He died to redeem. Certainly we have "pierced Him"; and then we shall see it. But, O fainting soul, to be "pierced" though in glory is the work for us of His real, His consoling kinship. "Behold and see," He says, "that it is I myself; I am He that liveth and was dead." . . .

O Blessed Master, keep us near Thee here, for then we cannot bear to be without Thee! Show us this life in the Light of Thy Coming; cleanse us from stains in the Fount of Thy Sorrows; bring us, soiled and struggling, to the Vision of Thy joy. . . .—*Canon Knox-Little.*

### Notes and Queries.

*Old Nurses.*

(71) Will you kindly tell me the name of the nursing association which was started to obtain employment for nurses too old for the ordinary nursing homes?—*A. C.*

Do you mean the Auxiliary Nurses' Society, 10 Orchard Street, W.? It is only open to members of the Royal British Nurses' Association.

*Maternity Training.*

(72) Can you tell me if there are any private maternity homes in Liverpool or Southport, where pupils are trained for a short period?—*A. H.*

We do not give the names of private institutions.

*Indulgence Passage.*

(73) Can you tell me anything about what is called an "indulgence passage" to South Africa? I have a friend in South Africa who tells me that a nurse can get an "indulgence passage" out, and I should be much obliged by any information.—*Zardi.*

"Indulgence passages" are granted to persons engaged in Government service. A nurse not so engaged would be ineligible. Assisted passages are granted to nurses, amongst others, by the South African Expansion Emigration Committee, 47 Victoria Street, Westminster, S.W. For particulars apply to the Secretary.

*Hospital Training.*

(74) I am 21 and anxious to become a nurse. Will you advise me what to do, and how to employ my time until I enter upon my training?—*Anxious One.*

Occupy your time in learning sick-cookery, elementary anatomy, and physiology, and you might also go to a children's hospital for a time.

*Indian Nursing Service.*

(75) With regard to the article published a few weeks ago about the appointment of 16 extra nurses for the Indian Nursing Service, can you tell me when the election takes place, or if they have already been elected?—*M. F.*

Apply to the India Office, St. James's Park, S.W.

*Registered Nurse.*

(76) Will you kindly tell me to whom I should apply in order to become a registered nurse?—*Anxious.*

There is no registration of nurses in this country.

*Warm Feet.*

(77) Will you kindly tell me of a treatment for the cure of hot feet?—*P. S. W.*

We do not prescribe treatment.

*Matron's Salary.*

(78) I should be much obliged if you could tell me what is the average amount paid in salary to the matron of a general hospital of 40 beds. I am the hon. secretary of a small hospital, and, in revising list of salaries, it would be of great assistance to know what is done in other institutions.—*H. V. H.*

The salary varies with the nature of the work, but £55 may be taken as an average sum.

*£1,000 Prize.*

(79) Will you tell me if you know anything about a prize of £1,000 offered for an improved bed-pan? A friend of mine in Australia has invented a pan and has heard of this offer, but she cannot find out anything more about it.—*R. A. M. C.*

We have heard nothing of it.

*How to Sterilize.*

(80) Will you kindly tell me the simplest and safest way to sterilize a new indiarubber syringe.—*Sister Dora.*

Wash well in soap and water, and rinse in clear water to which a disinfectant, of which there is a large choice, has been added.

**Important Nursing Textbooks.**

"The Nursing Profession: How and where to Train." 2s. net 2s. 4d. post free.

"A Handbook for Nurses." By Dr. J. K. Watson. 5s. net; 5s. 4d. post free.

"Practical Guide to Surgical Bandaging and Dressings." By Wm. Johnson Smith, F.R.C.S. 2s. post free.

"The Nurses' Dictionary of Medical Terms and Nursing Treatment." By Honnor Morten. 2s. post free.

"The Human Body: its Personal Hygiene and Practical Physiology." By B. P. Colton. 5s. post free.

"Art of Feeding the Invalid." (Popular Edition). 1s. 6d. post free.

"On Preparation for Operation in Private Houses. By Stanmore Bishop, F.R.C.S. 6d. post free.

## Figures and Tables

**Fig. 1. f1:**
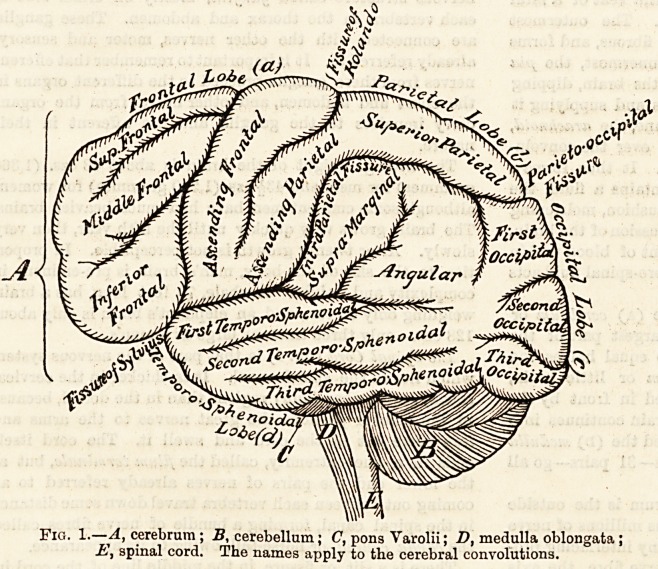


**Fig. 2. f2:**
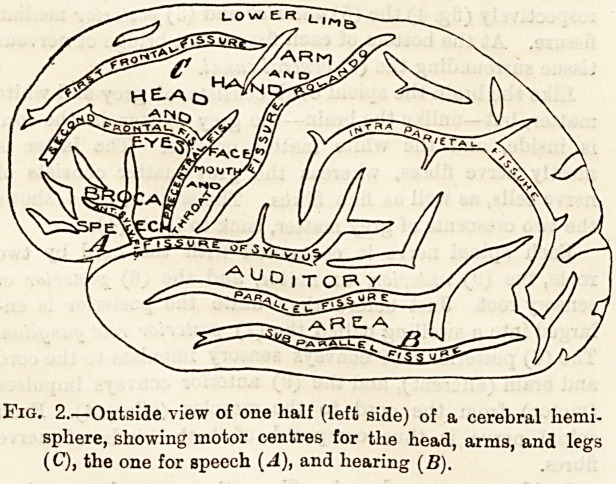


**Fig. 3. f3:**
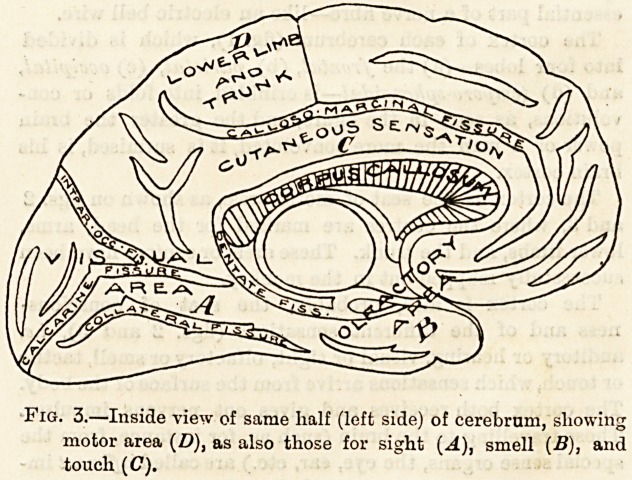


**Fig. 4. f4:**